# Boron nitride colloidal solutions, ultralight aerogels and freestanding membranes through one-step exfoliation and functionalization

**DOI:** 10.1038/ncomms9849

**Published:** 2015-11-27

**Authors:** Weiwei Lei, Vadym N. Mochalin, Dan Liu, Si Qin, Yury Gogotsi, Ying Chen

**Affiliations:** 1 Institute for Frontier Materials, Deakin University, 75 Pigdons Road, Waurn Ponds, Geelong, Victoria 3216, Australia; 2 A. J. Drexel Nanomaterials Institute, and Materials Science and Engineering Department, Drexel University, 3141 Chestnut Street, Philadelphia, Pennsylvania 19104, USA; 3 Department of Chemistry, Missouri University of Science & Technology, 1870 Miner Circle, Rolla, Missouri 65409, USA

## Abstract

Manufacturing of aerogels and membranes from hexagonal boron nitride (h-BN) is much more difficult than from graphene or graphene oxides because of the poor dispersibility of h-BN in water, which limits its exfoliation and preparation of colloidal solutions. Here, a simple, one-step mechano-chemical process to exfoliate and functionalize h-BN into highly water-dispersible, few-layer h-BN containing amino groups is presented. The colloidal solutions of few-layer h-BN can have unprecedentedly high concentrations, up to 30 mg ml^−1^, and are stable for up to several months. They can be used to produce ultralight aerogels with a density of 1.4 mg cm^−3^, which is ∼1,500 times less than bulk h-BN, and freestanding membranes simply by cryodrying and filtration, respectively. The material shows strong blue light emission under ultraviolet excitation, in both dispersed and dry state.

## Abstract (web summary)

The poor dispersibility of 2D hexagonal boron nitride in water currently limits its exfoliation and applications. Here, the authors present a one-step mechano-chemical process to achieve unprecedented colloidal concentrations, which permits fabrication of ultralight aerogels and freestanding membranes.

## Graphical abstract

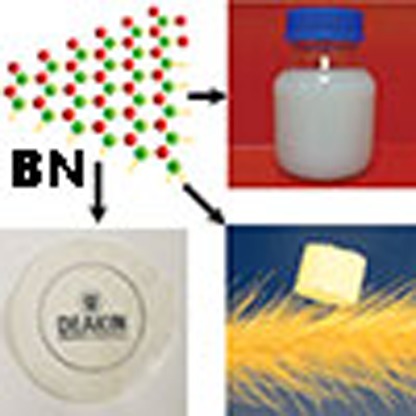

Two-dimensional boron nitride (BN) nanosheets, also called ‘white graphene' or ‘non-carbon graphene', consist of a few layers of alternating boron and nitrogen atoms in a hexagonal arrangement. Their mechanical, thermal and electronic properties are attractive for applications in polymer matrix composites^1^,^2^, hydrogen storage^3^, field emitters^4^, electrocatalysts^5^ and sorbents^6^,^7^. In addition, it has been demonstrated that BN single crystals, unlike carbon materials, have strong luminescence in the ultraviolet range, which makes BN attractive for blue light and ultraviolet emission^8^.

In recent times, significant efforts have been focused on the isolation and functionalization of BN nanosheets to achieve better dispersion, which would enable applications in optical devices, biological systems and composites^9^,^10^,^11^. Organic solvents such as N,N-dimethylformamide have been employed for exfoliation and stabilization of disperse BN nanosheets, through polar–polar interactions between the functional groups and the hexagonal BN (h-BN) surface, using a sonication–centrifugation technique^12^. Lin *et al.*^13^ have reported that ball-milled h-BN with an increased number of defects can be functionalized with a long alkyl chain amine via Lewis acid–base interactions to produce water-soluble sheet-like particles. They also reported that water dispersion of h-BN nanosheets can be achieved directly by using water as the exfoliating molecule through edge functionalization^14^. In recent times, Sainsbury *et al.*^15^ obtained hydroxyl-terminated BN nanosheets by the oxidation of exfoliated h-BN nanosheets using a two-step functionalization procedure. However, the concentration of the h-BN dispersions was typically below 2 mg ml^−1^, even after long periods of intense ultrasonication. The low concentration may present an especially severe limitation for the aqueous suspensions preferred in many applications. Therefore, the development of a practical high-yield process to achieve highly water-soluble BN nanomaterials remains a challenge.

Graphene oxide and graphene, in the form of aerogels and membranes^16^,^17^,^18^, have been used as efficient adsorbents for the separation of organic pollutants and oils from water^19^,^20^, for gas separation^21^ and for molecular and ion selective devices^22^,^23^. There is potential for h-BN, which is more oxidation and intercalation resistant than *sp*^2^ carbon, to be used to produce similar structures^24^,^25^,^26^. The h-BN-based materials represent a great advantage for high-temperature applications and extreme environments. However, it is very difficult to achieve aqueous dispersion of h-BN using conventional routes. In recent times, BN foams assembled from nanosheets showed excellent thermal stability, super-elasticity and very low electrical permittivity, but the assembly process required the use of templates, high temperature and vacuum, hazardous and toxic chemicals, and sophisticated fabrication techniques^27^,^28^.

Here we present a simple and efficient one-step method for the preparation and functionalization of few-layer BN by solid-state ball milling of commercially available h-BN and urea powder. This ambient temperature method has several advantages, including scalability for mass production, low cost, high yield and does not require the use of organic solvents, catalysts, substrates or vacuum systems. The as-produced few-layer BN shows unprecedentedly high dispersibility in water, yielding stable colloidal solutions with concentrations up to 30 mg ml^−1^. A urea-assisted exfoliation and functionalization mechanism is proposed to explain the formation of the water-soluble few-layer BN. Ultralight BN aerogels can be produced by cryodrying and thin freestanding membranes fabricated by filtration of the two-dimensional few-layer BN dispersions. Both colloidal solutions and freestanding membranes show strong ultraviolet and blue light luminescence.

## Results

### Synthesis and characterization

**Figure 1:**
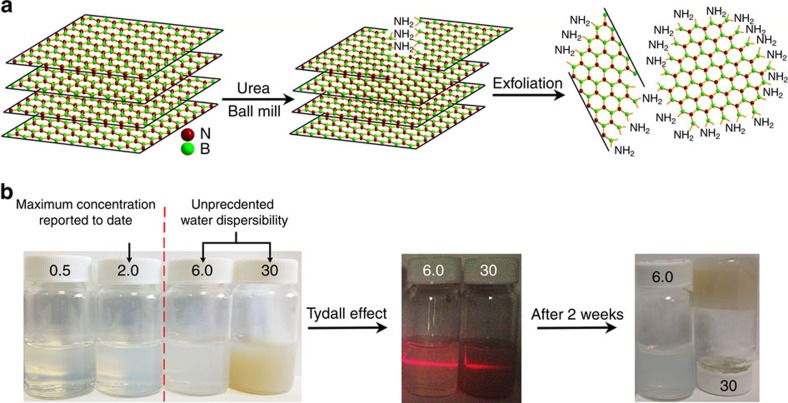
Schematic illustration of the exfoliation and dispersion process. (**a**) Schematic illustration of the exfoliation. (**b**) Photos of as-prepared colloidal solutions of few-layer BN with concentrations of 0.5, 2.0, 6.0 and 30 mg ml^−1^, respectively, and demonstration of the Tyndall effect and stability of the colloidal solutions.

We have developed a one-step method for the preparation and functionalization of few-layer BN based on urea-assisted solid exfoliation of commercially available h-BN (Fig. 1a). After ball milling and washing with water to remove urea, few-layer BN can be readily dispersed in water without sonication, to form stable colloidal solutions at different concentrations (Fig. 1b and Supplementary Fig. 1). The concentration of the suspensions can reach up to 30 mg ml^−1^ (Fig. 1b), the highest ever value reported for BN water suspensions^10^,^11^,^12^,^13^,^14^,^15^. The suspensions with a high concentration up to 30 mg ml^−1^ exhibited a milky and light yellow appearance compared with the low-concentration suspensions, possibly due to a relatively high concentration of amino groups^29^,^30^. The yield of the exfoliated BN sheets in the proposed urea-assisted, high-energy ball mill processing was as high as 85%. The path of a red laser beam can be clearly seen through all the dispersions due to light scattering by the few-layer BN colloid (Tyndall effect), as shown in Fig. 1b. The aqueous BN dispersion with the highest concentration (30 mg ml^−1^) transformed into a hydrogel within 2 weeks when left undisturbed (Fig. 1b). Based on the Doi and Edwards theory^31^, the sol-gel transformation can be attributed to the action of van der Waals interparticle forces at the contact points, which will lead to formation of a three-dimensional (3D) continuous network in highly concentrated nanosheet dispersions. Scanning electron microscopy (SEM) images show that the pristine non-milled h-BN has particles with lateral dimensions of 2–10 μm, with smooth surfaces and edges, which are stacked together (Supplementary Fig. 2a). In contrast, the dispersed particles have a fluffy and lamellar morphology. The images of these samples, formed by evaporating a drop of diluted aqueous suspension on a Si wafer can be seen in Supplementary Fig. 2b,c. The size and the thickness of the dispersed few-layer BN sheets are reduced compared with the pristine sheets.

**Figure 2:**
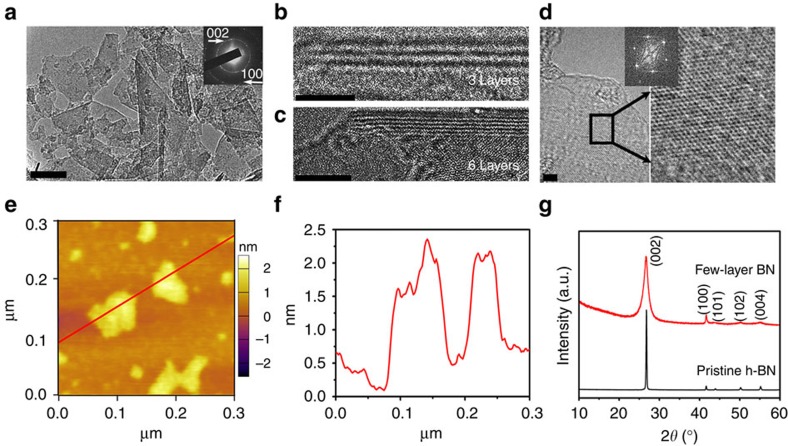
Exfoliated few-layer BN. (**a**) TEM image of few-layer BN, with the selected-area electron diffraction pattern (inset) indicating a layered BN structure. Scale bar, 50 nm. (**b**,**c**) HRTEM images of the edge folding of two few-layer BN sheets with three and six BN layers, respectively. Scale bars, 2 nm (**b**) and 5 nm (**c**). (**d**) HRTEM and the fast Fourier transform images of a few-layer BN sheet. Scale bar, 2 nm. (**e**,**f**) Atomic force microscopy image and corresponding line-scan profile of few-layer BN. (**g**) XRD patterns of few-layer BN and pristine h-BN.

Figure 2a shows the transmission electron microscopy (TEM) image of the few-layered BN prepared by evaporating a drop of diluted colloidal solution on a carbon-coated copper grid. The TEM image suggests that the BN particles are flat and quite thin. The selected area electron diffraction pattern shows two characteristic diffraction rings ((002) and (100)) of layered BN (Fig. 2a inset). The selected area electron diffraction image has a slightly pronounced hexagonal pattern, due to the few-layer stacking of h-BN. The high-resolution TEM (HRTEM; Fig. 2b,c) images at the edges of few-layered BN clearly show three and six parallel fringes corresponding to three and six stacked layers in the samples. As seen in the HRTEM and the fast Fourier transform images in Fig. 2d, the structure of the few-layer BN remains ordered after ball milling. The thickness and lateral size distributions were statistically evaluated by HRTEM measurements on 100 sheets (Supplementary Fig. 3). Eighty-three per cent of the BN sheets were <2.5 nm thick and around 100 nm in lateral size. The nanometre-scale thickness of few-layered BN was further confirmed by atomic force microscopy (Fig. 2e). Figure 2f suggests that the thickness of the few-layer BN is about 2 nm (five to six monolayers). The structure of few-layer BN was further investigated by X-ray diffraction (XRD) analysis. As shown in Fig. 2g, two main characteristic diffraction peaks can be observed at 26.2° and 42.8°, arising from (002) and (100) planes of few-layer BN, respectively. Compared with the pristine h-BN, the (002) and (100) peaks of few-layer BN show a remarkably reduced intensity and dramatically broadened width, indicating the presence of thin BN sheets and much less extended/ordered stacking in the *c* direction.

The Fourier transform infrared (FTIR) spectrum shows an additional peak at 3,240 cm^−1^ due to the N–H stretching vibration^10^,^32^, whereas pristine h-BN exhibits only the characteristic peaks of in-plane B–N stretching vibrations at ∼1,364 cm^−1^ and out-of-plane B–N–B bending vibrations at ∼1,746 cm^−1^ (Supplementary Fig. 4)^33^. The shoulder at 3,411 cm^−1^ can be ascribed to O–H stretching vibrations due to the washing in water after ball milling^10^,^14^,^15^. The appearance of N–H stretching vibrations indicates that functionalization has occurred. Functional groups such as NH_2_ from urea have been created and bonded to the defect sites and edges of few-layer BN. In addition, the strong pungent smell and the readings of an ammonia sensor on opening the milling jar further demonstrate that ammonia gas and/or low-molecular-weight amines were formed as intermediates in the mechano-chemical process. Although the recently reported hydroxyl functionalized BN nanosheets prepared via NaOH-assisted ball milling had very low dispersibility in water with highest achievable concentrations <1 mg ml^−1^ (ref. 11), the functionalized few-layer BN reported here forms aqueous colloidal solutions with concentration up to 30 mg ml^−1^.

Dispersions of the pristine BN and few-layered BN were investigated using zeta potential measurements^34^. Both of them exhibit highly negative potential values, −44±3 and −34±4 mV, respectively, consistent with the reported values for BN materials due to the B–O–H and N–O–H generated on h-BN in water^35^,^36^. It has been reported that the hydrophilicity, shape, size distribution and zeta potential of particles are all important factors governing their dispersibility in water and stability of formed dispersion^35^,^36^. The excellent hydrophilicity and narrow size distribution are essential to the good dispersibility, whereas high zeta potential helps to stabilize the dispersion. Although zeta potential of pristine BN is −44±3 mV, it cannot disperse in water well due to its hydrophobicity and large particle size (Supplementary Figs 5a and 2a)^35^. The few-layer BN exhibits a somewhat decreased absolute value of zeta potential (−34±4 mV) after functionalization with NH_2_ groups. The reduced negative zeta potential after functionalization with NH_2_ groups is due to partial neutralization effect between positively charged NH_3_^+^ groups and negatively charged oxygen-containing groups formed on BN surface on contact with water. Thus, although the absolute value of zeta potential decreased compared with pristine BN, it is still quite high and the few-layer BN demonstrates excellent water dispersibility compared with pristine BN. In addition to electrostatic stabilization, this result is attributed to the improved hydrophilicity, uniform thickness and a narrower size distribution after ball milling and the attachment of NH_2_ groups (Fig. 2 and Supplementary Fig. 5b).

The high-resolution B 1s and N 1s X-ray photoelectron spectroscopy (XPS) results are shown in Supplementary Fig. 6. In Supplementary Fig. 6a, the component at 190.4 eV corresponds to B–N bonds in h-BN and the shoulder (191.5 eV) should be attributed to the B atoms in B–O bonds formed due to the exposure of the few-layered BN to ambient environment and the hydroxyl groups attached to defects along the edges of h-BN during washing in water after ball milling^14^,^37^. In addition, the B–O bonds may originate from the pristine h-BN. In the synthesis process of h-BN, oxygen-containing boron trioxide (B_2_O_3_) or boric acid are typically used as a precursor. It is possible that the h-BN particles used in the present study contained small amounts of oxygen. In Supplementary Fig. 6b, the main peak with a binding energy of 398.2 eV in the N 1s spectrum corresponds to N–B bonds in h-BN; a shoulder can be deconvoluted at 399.6 eV corresponding to N–H bonds, which is another evidence of the presence of amino group^37^. The amount of the attached NH_2_ groups was evaluated from the thermogravimetric analysis (TGA), which suggests ∼1.3 wt% content of NH_2_ groups in the functionalized h-BN sheets (Supplementary Fig. 7). The presence of urea-induced N–H-containing groups render the few-layer BN hydrophilic, with an average observed contact angle of 43±1° resulting in a high dispersibility in water, whereas the pristine BN has an average contact angle of 115±4° (Supplementary Fig. 5). Thus, NH_2_ species produced from urea and attached to BN during ball milling are strongly beneficial to the colloidal properties of the few-layer BN, providing stable aqueous dispersions. To confirm this, the pristine h-BN was milled with NaCl, which has previously been used to de-agglomerate nanodiamond^38^, under the same milling conditions. Compared with the sample milled with urea, there was no apparent water solubility of h-BN after milling with NaCl (Supplementary Fig. 8). This shows the importance of the presence of urea in the mechano-chemical process of exfoliation and functionalization of BN, yielding hydrophilic few-layer BN readily dispersible in water.

### Theoretical simulations

**Figure 3:**
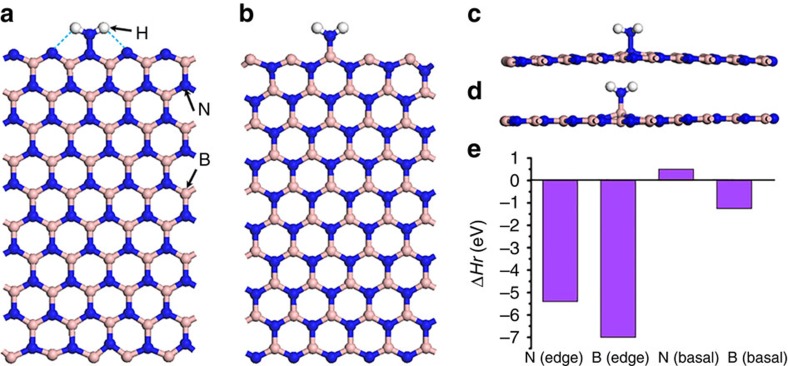
Optimized configurations of BN–amine interaction. (**a**) An NH_2_ at the nitrogen edge atom, (**b**) an NH_2_ at the boron zigzag edge atom, (**c**) an NH_2_ on a basal-plane N atom and (**d**) an NH_2_ on a basal-plane B atom. (**e**) Reaction enthalpies for the different optimized geometries of the configurations (**a**–**d**).

**Figure 4:**
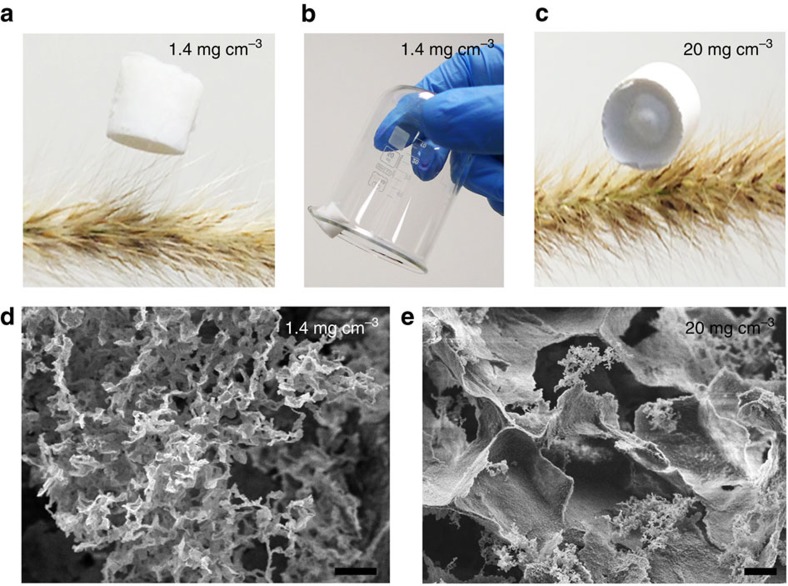
BN aerogel. (**a**) Photo of an aerogel with a low density (1.4 mg cm^−3^) placed on the spike of a plant. (**b**) Photo of an aerogel adhering to a beaker wall. (**c**) Photo of an aerogel of a higher density (20 mg cm^−3^) pressing the spike of a plant. (**d**,**e**) SEM images of aerogels with densities of 1.4 and 20 mg cm^−3^, respectively. Scale bars, 2 μm (**d**,**e**).

Interactions of amino groups with an h-BN monolayer were studied by density functional theory (DFT) calculations. The following sites for the adsorption of NH_2_ on the h-BN single layer were considered: (1) N atoms at the zigzag edge of BN (Fig. 3a), (2) B atoms at the edge of BN (Fig. 3b), (3) basal-plane N atoms (Fig. 3c) and (4) basal-plane B atoms (Fig. 3d). The corresponding bond lengths and angles, as well as final enthalpy values for the most stable structures of each configuration produced by geometry optimization, are listed in Supplementary Table 1.

As expected, due to the dangling bond saturation, the structures where an NH_2_ group is attached to the zigzag edge of h-BN (Fig. 3a,b) are more stable than the structures where the NH_2_ is attached to the atoms in the basal plane (Fig. 3e). For NH_2_ attached to an edge N atom (Fig. 3a), there is evidence of additional interactions between H atoms of the amino group and the nearby N atoms, potentially hydrogen bonding, indicated by dashed lines in Fig. 3a. This is supported by an unusually short N–N distance between the N atom of the NH_2_ and the edge N atom of h-BN, as well as a large H–N–H valence angle of NH_2_ (Supplementary Table 1). These interactions additionally stabilize the structure. Still, bonding of NH_2_ to B terminating the edge resulted in the most stable and preferred configuration of the four considered, as shown by the enthalpy of reaction (Fig. 3e).

For both structures with basal attachment of NH_2_ (Fig. 3c,d), by contrast, the formed bonds are longer (Supplementary Table 1), an indication of steric hindrance to NH_2_ bonding. The basal B atom bonded to NH_2_ comes slightly off the BN plane, signifying its transition from planar to tetrahedral bonding. The configuration with NH_2_ attached to a basal N atom of h-BN is the least energetically stable (Supplementary Table 1) and its formation is unfavourable, as indicated by a positive Δ*H*_r_ value (Fig. 3e). The H–N–H angle of NH_2_ in this configuration (Fig. 3c) is close to unbonded NH_2_ (Supplementary Table 1) and the HNH plane is oriented more in parallel to the h-BN plane in contrast to its nearly perpendicular orientation in another basal configuration (Fig. 3d). These are indicators that NH_2_ adsorption, not covalent bonding, may be the most probable mechanism in this case. In fact, we observed that in several simulations when the NH_2_ group was not precisely positioned above the basal N atom, it moved and formed covalent bond with a neighbouring B atom, ending up in a configuration similar to Fig. 3d. Thus, DFT modelling confirms that NH_2_ groups can be adsorbed and chemically bonded to h-BN. Most preferred sites for chemical bonding are the edge B and N atoms, with covalent bonding to the edge B atom being the most favourable. On the basal BN plane NH_2_ bonding to a B atom is possible, whereas the reaction of NH_2_ with a basal N atom is not energetically favourable.

### Mechanism of exfoliation and functionalization of h-BN

Based on the above structural characteristics and analysis, as well as DFT calculations, we propose the following mechanism for the one-step exfoliation and functionalization of few-layer BN. The exfoliation process involves two stages.

At the beginning of ball milling, the sizes of pristine h-BN and urea particles are reduced due to the high shear forces and high-energy collisions with the stainless steel balls. In this process, BN and urea particles are thoroughly mixed together and some small urea particles adsorb on the BN surface. The layered BN structure is wedged by the small urea particles, resulting in a slight increase of the interlayer spacing (Supplementary Fig. 9). Some urea molecules may intercalate into the BN structure from the edges of BN layers during high-energy ball impacts. Size reduction and exfoliation were confirmed by the XRD patterns of the products after 20 h milling (Supplementary Fig. 9) without washing with water, which show dramatically broadened and less intense (002) diffraction. An excess of urea over BN ensured sufficient intercalation of urea molecules into the BN structure^39^,^40^,^41^.

Mechano-chemical processes powered by the energy of colliding balls lead to decomposition of urea and chemical bonding of NH_2_ groups to few-layer BN^42^. This results in the attachment of NH_2_ to BN edges and defects, as confirmed by FTIR and XPS spectra and TGA (Supplementary Figs 4, 6 and 7), and prevents restacking of BN sheets. The DFT calculations demonstrate that the NH_2_ groups can be adsorbed on the B sites of the BN surface and easily form bonds with the edge B and N sites, rendering few-layer BN hydrophilic and assisting in its stabilization in aqueous dispersions.

### BN aerogels and membranes

Functional aerogels and transparent thin membranes^16^,^17^,^18^ have been extensively developed, to exploit the properties of nanomaterials over a wide range of applications. Ultralight BN aerogels and freestanding membranes can be produced via cryodrying and filtration of few-layer BN suspensions, respectively. A BN aerogel can be easily prepared by a one-step cryo-desiccation of the BN aqueous dispersion (Fig. 4). The ultralow density of the aerogel was demonstrated by placing it on the delicate spike of a plant (Fig. 4a), as well as adhering to a nearly vertical beaker wall by electrostatic attraction (Fig. 4b). The density of the aerogel was estimated, including the density of the air occupying the pores, to be in the range from 1.4 to 20 mg cm^−3^, depending on the concentration of few-layer BN in the aqueous dispersion. The 1.4 mg cm^−3^ BN aerogel is the lightest reported among BN-based materials and much lighter than many carbon and other aerogels^43^,^44^,^45^,^46^,^47^. In addition, the Brunauer–Emmett–Teller analysis of low-temperature nitrogen adsorption isotherms shows that the BN aerogel has a high specific surface area of 273 m^2^ g^−1^ (Supplementary Fig. 10). Barret–Johner–Halenda calculations give a broad pore size distribution in the range of 2–50 nm. Larger pores were probably present, but they could not be measured by gas adsorption. The heavier sample, with a density of 20 mg cm^−3^, distorted the seed spike more (Fig. 4c). The corresponding SEM investigations revealed that the BN low-density aerogel has a loose 3D structure of pores connected by thin walls, whereas the higher density aerogel has larger ‘sheet-like' structure of thicker connecting walls (Fig. 4d,e). The ultralow weight together with low electrical conductivity and high thermal and chemical stability of BN enable potential applications of these gels in insulation, dielectrics and ultralight materials^24^.

**Figure 5:**
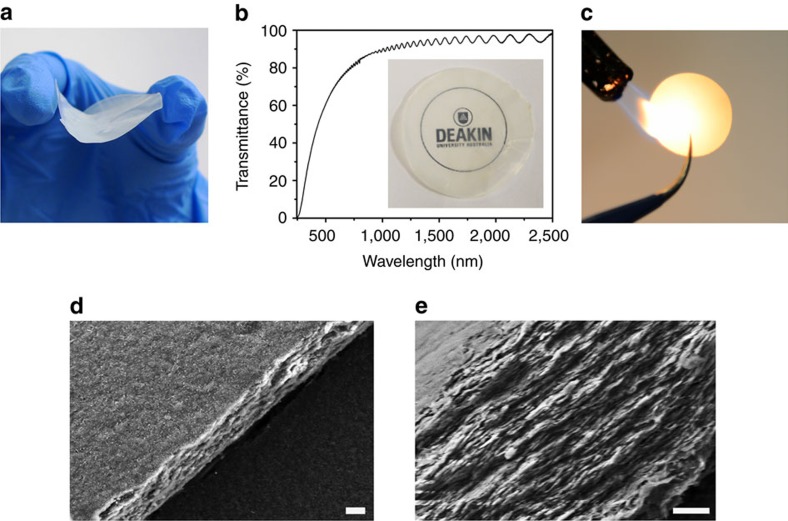
BN membranes. (**a**) Photo of the freestanding BN membrane. (**b**) Optical transmittance of the freestanding BN membrane and inset is a photo of the BN membrane placed over a paper with a printed logo. (**c**) Photo of a BN membrane held in the flame in ambient air. SEM images of the basal (**d**) and fracture surface (**e**) of the BN membrane. Scale bars, 2 μm (**d**) and 1 μm (**e**).

BN membranes were directly fabricated using vacuum-assisted filtration of the few-layer BN dispersion. The membrane can be readily peeled off from the filter and retains its mechanical integrity, while being flexible in the freestanding state (Fig. 5a). This ultrathin membrane is nearly transparent to visible light as shown in Fig. 5b. The optical transparency of the membrane can be attributed to its thickness and the inherently low extinction coefficient of BN in the visible range^48^—in striking contrast to graphene. Furthermore, the thickness of the membrane can be tuned in the range of 0.2–100 μm by filtering the desired quantity of few-layer BN dispersion with a fixed concentration. The optical transmittance of a freestanding membrane (∼10 μm thick according to SEM) was measured by ultraviolet–visible–near infrared spectrometer and is shown in Fig. 5b. The membrane completely absorbs ultraviolet light, while retaining high optical transmittance in the visible range above 500 nm and up to 95% transmittance in the near-infrared range. The oscillations in the transmittance in the near-infrared region are a result of the interference of light reflected at the air–membrane and membrane–substrate interfaces. The pronounced and clean interference pattern indicates a relatively smooth surface and uniform thickness of the membrane^49^. These optical properties indicate potential applications of BN membranes for ultraviolet shielding/protection. Moreover, the BN membrane is also fire-resistant and does not burn in the flame of a gas lighter in ambient air (Fig. 5c). BN membranes are much more thermally stable and have a higher resistance to oxidation than any carbon-based material. Figure 5d,e show the top surface and cross-sectional SEM images of the BN membrane. The cross-sectional image, as shown in Fig. 5e, reveals a compact stacking of few-layer BN with uniform thickness (∼10 μm).

### Photoluminescence spectra

**Figure 6:**
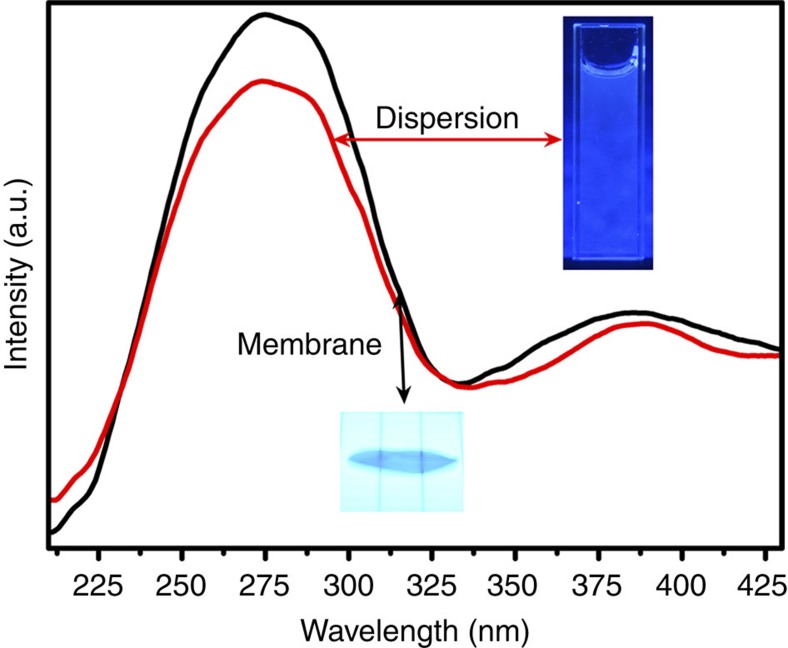
Photoluminescence of BN. Emission spectra of colloidal solution and a BN membrane under excitation at 200 nm. The insets are the corresponding photos.

Figure 6 shows the photoluminescence (PL) spectra of a few-layer BN dispersion in water and of a freestanding membrane recorded with an excitation wavelength of 200 nm. As shown in Fig. 6, a strong ultraviolet PL emission peak at 275 nm can be seen in both samples, attributed to deep levels in the band gap from intrinsic B or N defects, which is similar to previously reported results for BN nanosheets, bamboo-like multiwall nanotubes and nanohorns containing bent BN layers^50^,^51^,^52^. There is a weak peak at ∼385 nm, appearing as a shoulder to the main PL peak. This peak may be assigned to impurities (possibly oxygen) or to generic structural defects^53^,^54^,^55^,^56^. The digital photographs (Fig. 6 insets) of the aqueous dispersions and freestanding membranes under 365 nm ultraviolet light further confirm the blue light emission. The strong ultraviolet and blue light emission indicates that few-layer BN dispersions and freestanding membranes may have potential applications in bioimaging, ultraviolet laser emitters and optoelectronic devices.

## Discussion

A simple and high-yield process has been developed to fabricate few-layer h-BN with a thickness around 2.5 nm and lateral dimensions mostly below 100 nm. The procedure involves urea-assisted mechanical exfoliation during which mechano-chemical reactions take place at atmospheric pressure and room temperature. The residual urea can be washed away, resulting in a high concentration of well-dispersed few-layer BN in water without any posttreatment. Ultralight and freestanding aerogels can be produced by cryodrying of the few-layer dispersions, whereas filtration gives thin, high-quality membranes of controllable thickness. Importantly, the as-obtained few-layer BN dispersions and membranes show strong light emission when excited with an ultraviolet source. The stable colloidal aqueous dispersions of few-layer BN, ultralight aerogels and freestanding membranes produced in this study may be highly beneficial for a wide range of applications.

## Methods

### Synthesis

In a typical synthesis, h-BN (Momentive Performance Materials Inc.) and urea (Sigma-Aldrich) were mixed together at the weight ratio 1:60 inside a steel milling container using a planetary ball mill (Pulverisette 7, Fritsch) at a rotation speed of 700 r.p.m. for 20 h at room temperature under nitrogen atmosphere. The high rotation speed provides high power and effective exfoliation of h-BN on a large scale. The urea not only assists the exfoliation but also protects the BN from excessive mechanical damage, preventing an extensive formation of lattice defects. The variations in weight ratio (1:20 and 1:100) and milling time (10 and 30 h) were also investigated. The size and thickness of the few-layer BN particles were reduced at lower h-BN:urea weight ratio or longer milling time. The results are shown in Supplementary Table S2. In this study, the weight ratio of h-BN:urea and milling time were fixed at 1:60 and 20 h, correspondingly, to get BN sheets with suitable size and thickness for the following preparation of aerogel and membrane. After ball milling, the obtained powders were dissolved in water. The resulting few-layer BN aqueous dispersion was dialysed for 1 week (membrane cutoff: 3,500 kDa) in de-ionized water, to remove the urea. Stable aqueous dispersions were then obtained. For milling of pristine h-BN with NaCl, the same milling conditions were used. XRD and FTIR spectra were recorded to confirm the absence of any traces of urea and NaCl in the few-layer BN. The as-prepared hydrogel was directly dehydrated via a freeze-drying process to maintain the 3D monolith architecture. The BN membrane can be readily fabricated by vacuum filtration of a stable few-layer BN in water suspension through an Anodisc membrane filter (47 mm diameter, 0.02 μm pore size, Whatman), followed by drying in air and peeling the membrane off the filter.

### Materials characterization

XRD measurements were performed on a Panalytical X'Pert PRO apparatus with Cu Kα radiation. SEM analysis was performed on a Zeiss Supra 55 VP SEM instrument. The BN samples were sputtered with carbon before imaging. TEM and HRTEM imaging was performed on a JEOL 2100F microscope operated at 200 kV. Samples were prepared by evaporating a drop of diluted aqueous suspension on a carbon-coated copper grid. The atomic force microscopy measurements were performed on a Cypher atomic force microscope. The FTIR and optical transmittance spectra were recorded using a Nicolet 7199 FTIR spectrometer and Cary 5000 spectrophotometer, respectively. XPS was performed using an ESCALAB 250 instrument equipped with a non-monochromatic Mg-Kα X-ray source. The thermal behaviour was analysed by TGA on a TA Instruments Q50 TGA thermal analyser at a heating rate of 10 °C min^−1^ from room temperature to 800 °C under nitrogen gas flow. The zeta potential was measured using a Zetasizer Nano ZS90 apparatus from Malvern. The density of BN aerogel was determined by measuring the weight and volume of the aerogel. Nitrogen adsorption and desorption isotherms were obtained using a Tristar 3000 apparatus at 77 K. Contact angle measurements were made by a contact angle goniometer (CAM101, KSV). Contact angle results are presented as average of five to ten measurements at different locations±s.d. The emission spectra were recorded on a Fluoromax-4 spectrometer (Horiba Jobin Yvon Inc.).

### Theoretical simulations

The analysis of the different geometries was carried out using a pseudopotential plane-wave method within the DFT framework by Accelrys Materials Studio package. The DFT calculations were performed in the generalized gradient approximation with the Perdew–Burke–Ernzerhof functional implemented in CASTEP module of Accelrys Materials Studio. The BN nanoribbon infinite in one direction (labelled c) was constructed using Materials Studio Visualizer. First, a 5,5 BN periodic single wall nanotube was created and the lattice angles were modified from *α*=*β*=90°, *γ*=120° to *α*=*β*=*γ*=90°. The structure was then converted into a 1 × 1 × 5 (*a* × *b* × *c*) supercell. The nanotube was cut by manually removing a row of B–N bonds along the *b*-direction of the cell. The dimensions of the cell were increased to 22.0 × 22.00 × 12.75 Å (*a* × *b* × *c*), followed by quick geometry optimization in the FORCITE module with Dreiding forcefield, yielding a flat BN ribbon formed of 9 × 5 fused BN hexagonal rings, infinite in the *c*-direction and with zigzag edges. N atoms were exposed on one edge and B atoms exposed on the opposite. The cell dimensions were large enough to guarantee no interactions between the system and its periodic images, as the separation between any two atoms in the cell and their adjacent images was always larger than 10 Å. Finally, the geometry of an h-BN nanosheet prepared from periodic cells was carefully optimized with CASTEP. Following this, five cells of the same geometry and dimensions (22.00 × 22.00 × 12.75 Å), containing h-BN terminated by NH_2_ groups on the edge and basal B and N atoms, as well as a cell with just one NH_2_ group in the centre, were constructed.

Geometry of all systems was optimized using same settings in CASTEP (no cell optimization, energy cut-off 250.0 eV, 1 × 1 × 2 *k*-points on Monkhorst–Pack grid) with convergence tolerance 1.0 × 10^−5^ eV per atom (energy), 0.03 eV Å^−1^ (force), 0.05 GPa (stress) and 0.001 Å (displacement). The final enthalpy calculated by CASTEP was used as a measure of stability of the optimized structures. The enthalpy of reaction of an NH_2_ group with BN ribbon was calculated as 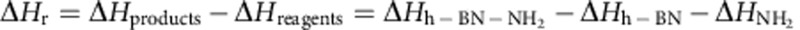, where 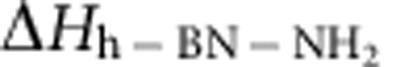 is the final enthalpy of an h-BN–NH_2_ structure, Δ*H*_h−BN_ and 
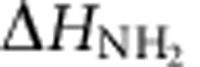
 are final enthalpies of h-BN nanoribbon and NH_2_ group placed in a periodic box of same dimensions and calculated using same settings, respectively.

## Additional information

**How to cite this article:** Lei, W. *et al.* Boron nitride colloidal solutions, ultralight aerogels and freestanding membranes through one-step exfoliation and functionalization. *Nat. Commun.* 6:8849 doi: 10.1038/ncomms9849 (2015).

## Supplementary Material

Supplementary Information Supplementary Figures 1-11 and Supplementary Tables 1-2
