# Supplementary material for: Boron nitride colloidal solutions, ultralight aerogels and freestanding membranes through one-step exfoliation and functionalization
Source: Nat Commun. 2015 Nov 27;6:8849. doi: 10.1038/ncomms9849 (PMC4674780; doi:10.1038/ncomms9849)
Supplement: Supplementary Information — Supplementary Figures 1-11 and Supplementary Tables 1-2 [file ncomms9849-s1.pdf]

Supplementary Figures

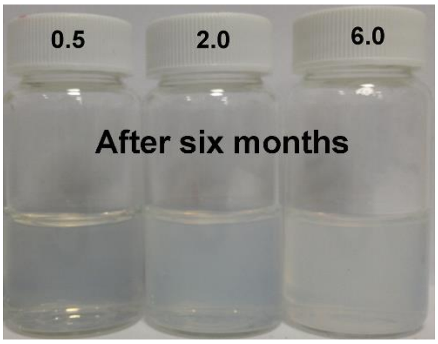

Supplementary Figure 1 | Photos of few-layer BN dispersions. Photos of as-prepared few-layer BN dispersions after 6 months with concentrations of 0.5, 2.0 and 6.0 mg ml^{-1}, respectively.

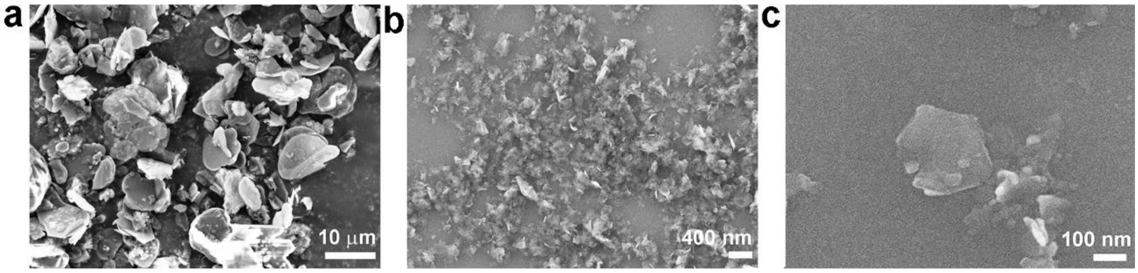

Supplementary Figure 2 | SEM images. a,**b**, Low-magnification SEM image of pristine h-BN and few-layer BN, respectively. **c**, High-magnification SEM image of few-layer BN.

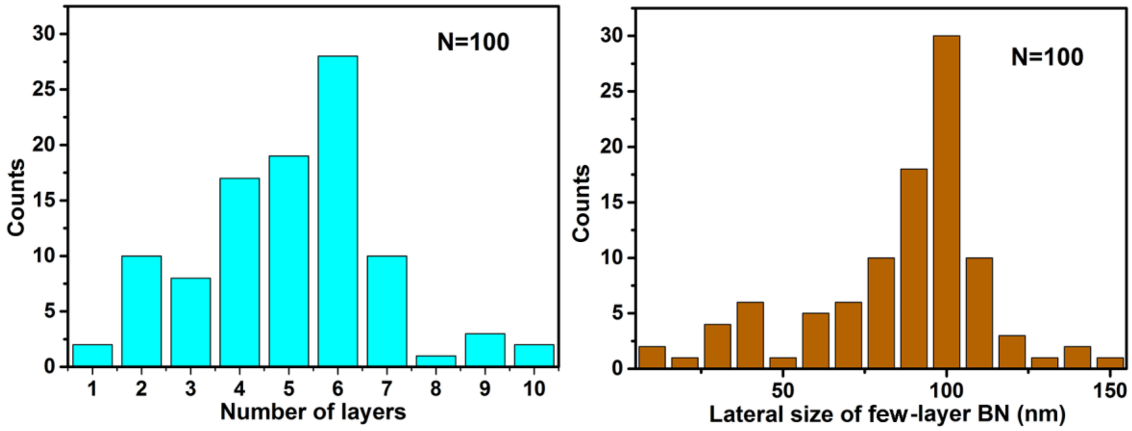

Supplementary Figure 3 | Statistical analysis of size distribution of few-layer BN. Statistical analysis of the number of thickness and lateral size for 100 BN sheets.

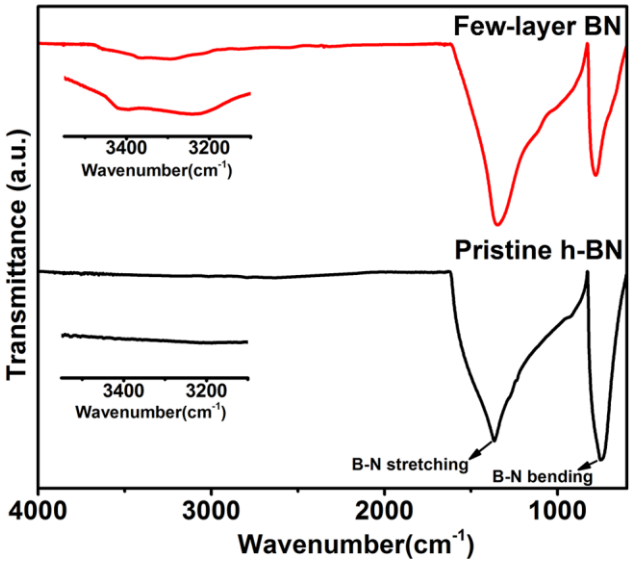

Supplementary Figure 4 | FTIR spectra. FTIR spectra of few-layer BN and pristine h-BN.

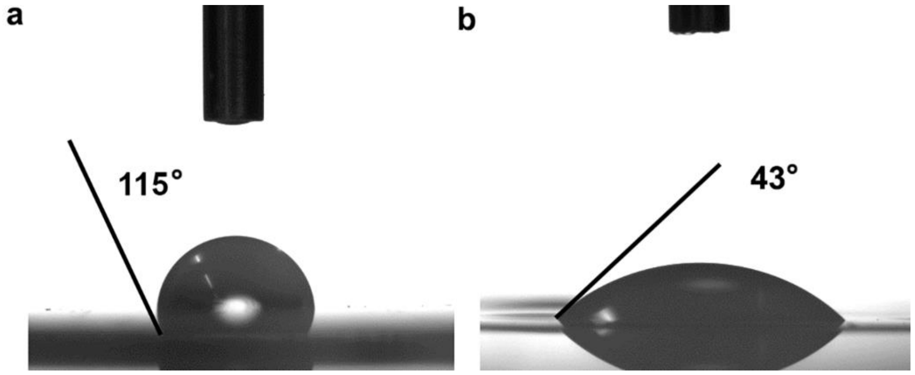

Supplementary Figure 5 | Contact angle measurements. **a**, Photograph of water droplet on pristine BN. **b**, Photograph of water droplet on few-layer BN.

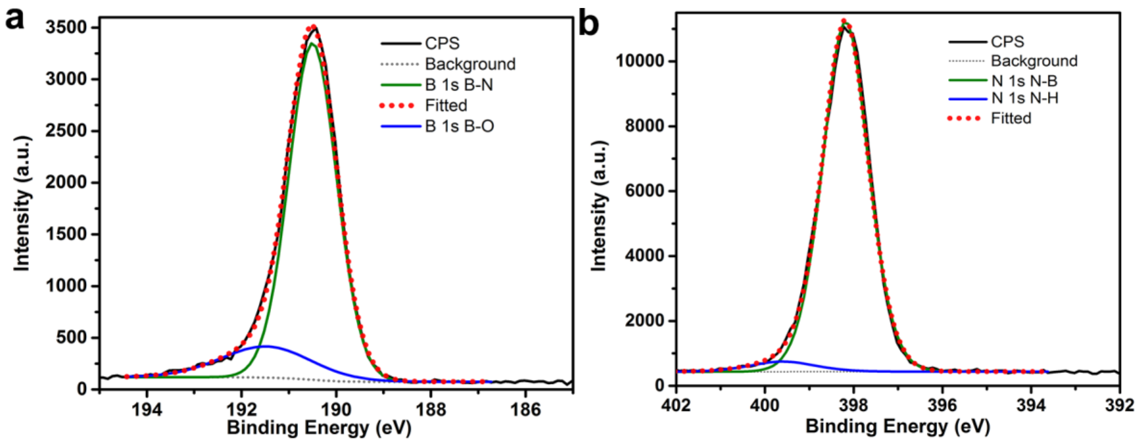

Supplementary Figure 6 | XPS spectra. XPS spectra of the few-layer BN: **a**, Core-level B1s. **b**, Core-level N1s.

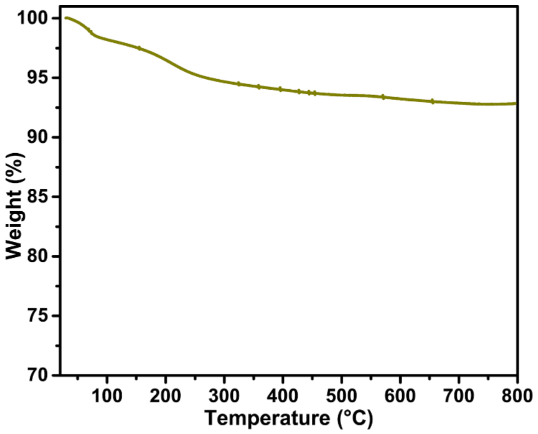

Supplementary Figure 7 | TGA curve of few-layer BN.

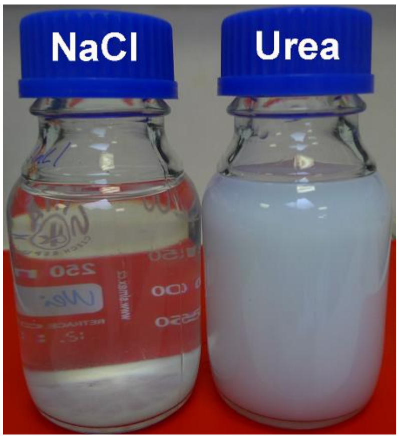

Supplementary Figure 8 | Optical Photo. Photo of h-BN dispersions in water after ball milling with NaCl and urea.

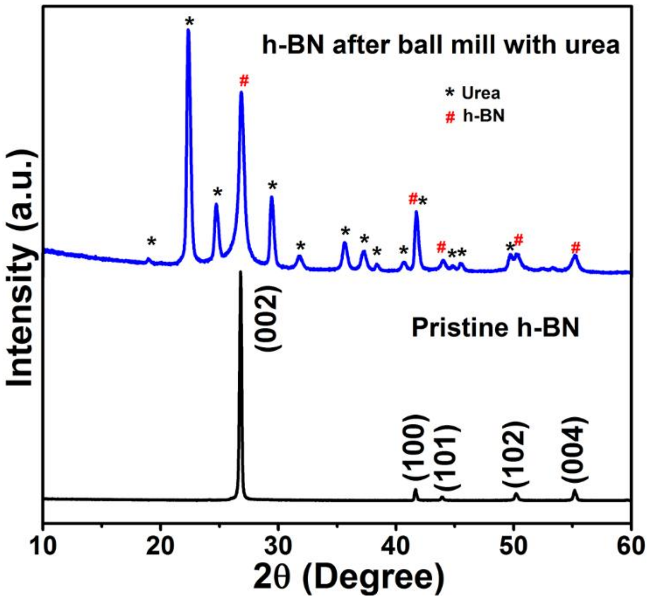

Supplementary Figure 9 | XRD patterns. XRD patterns of h-BN after 20 h milling with urea without washing with water.

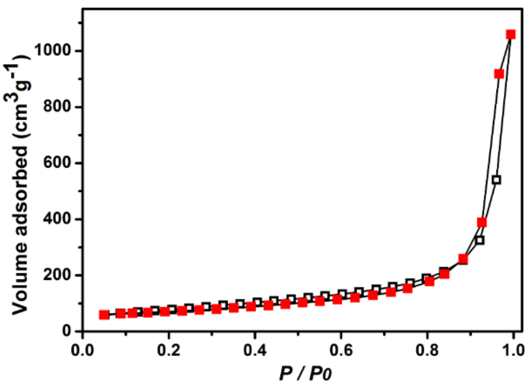

Supplementary Figure 10 | Nitrogen adsorption–desorption isotherm measurement of BN aerogel.

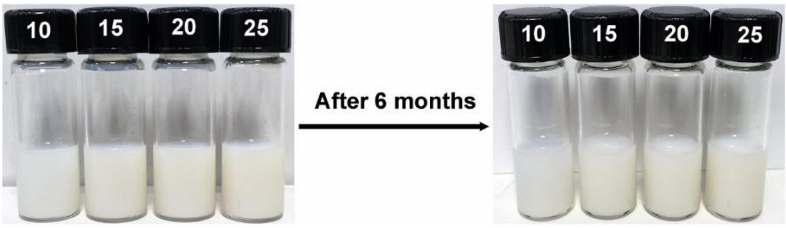

Supplementary Figure 11 | Photos of few-layered BN dispersions. Photos of as-prepared few-layer BN dispersions before and after 6 months of storage with concentrations of 10, 15, 20 and 25 mg ml^{-1}, respectively.

Supplementary Tables:

Structure	Bond distance, Å			∠HNH in NH_2, °	Final enthalpy, keV
	N-H in NH_2	B-N	N-N		
NH_2 attached to edge N atom	1.032 (1.033)		1.323	131	-17.808
NH_2 attached to edge B atom	1.023 (1.022)	1.396		115	-17.810
NH_2 attached to basal N atom	1.035 (1.035)		1.536	107	-17.802
NH_2 attached to basal B atom	1.027 (1.028)	1.556		110	-17.804
h-BN nanoribbon					-17.503
NH_2	1.045 (1.044)			103	-0.300

Supplementary Table 1 | Bond lengths, bond angles and final enthalpy energies for the optimal geometries.

Milling time / Ratio (h-BN : Urea)	10 h	20 h	30 h
1 : 20		Thickness:1-5 nm, Size: 100-200 nm	
1 : 60	Thickness: 5-10 nm, Size: 100-400 nm	Thickness:1-3 nm, Size: 50-100 nm	Thickness:1-2 nm, Size: 10-50 nm
1 : 100		Thickness:1-2 nm, Size: 5-50 nm	

Supplementary Table 2 | The effect of h-BN:urea ratio and ball milling time to the thickness and the lateral size of the functional BN sheets.
